# Clinical evaluation of two different protein content formulas fed to full-term healthy infants: a randomized controlled trial

**DOI:** 10.1186/s12887-018-1046-6

**Published:** 2018-02-13

**Authors:** Liotto Nadia, Orsi Anna, Menis Camilla, Piemontese Pasqua, Morlacchi Laura, Condello Chiara Cristiana, Giannì Maria Lorella, Roggero Paola, Mosca Fabio

**Affiliations:** 1 Fondazione I.R.C.C.S. Ca Granda Ospedale Maggiore Policlinico, Neonatal Intensive Care Unit Department of Clinical Science and Community Health, University of Milan, Milan, Italy; 2 Centro di Nutrizione a Partenza neonatale, Clinica Mangiagalli, Via Della Commenda, 12, 20122 Milan, Italy

**Keywords:** Low-protein formula, Safety, Growth, Body composition, Full-term infants

## Abstract

**Background:**

A high early protein intake is associated with rapid postnatal weight gain and altered body composition. We aimed to evaluate the safety of a low-protein formula in healthy full-term infants.

**Methods:**

A randomized controlled trial was conducted. A total of 118 infants were randomized to receive two different protein content formulas (formula A or formula B (protein content: 1.2 vs. 1.7 g/100 mL, respectively)) for the first 4 months of life. Anthropometry and body composition by air displacement plethysmography were assessed at enrolment and at two and 4 months. The reference group comprised 50 healthy, exclusively breastfed, full-term infants.

**Results:**

Weight gain (g/day) throughout the study was similar between the formula groups (32.5 ± 6.1 vs. 32.8 ± 6.8) and in the reference group (30.4 ± 5.4). The formula groups showed similar body composition but a different fat-free mass content from breastfed infants at two and 4 months. However, the formula A group showed a fat-free mass increase more similar to that of the breastfed infants. The occurrence of gastrointestinal symptoms or adverse events was similar between the formula groups.

**Conclusions:**

Feeding a low-protein content formula appears to be safe and to promote adequate growth, although determination of the long-term effect on body composition requires further study.

**Trial registration:**

The present study was retrospectively registered in ClinicalTrials.gov (trial number: NCT03035721 on January 18, 2017).

## Background

Increasing evidence indicates that early life represents a critical time window in terms of developmental programming [1]. A strict relationship between early growth pattern and later health outcomes has been reported, implicating nutrition as the underlying mechanism [2].

Breastfeeding is recommended as the normative standard for infants’ nutrition. Breastfed infants are adapted to a relatively high-fat, low-protein diet, which allows adequate growth without a concurrent increased risk for later adverse health outcomes [3]. Previous studies have demonstrated that non-breastfed infants who are fed a high protein formula during the first year of life show rapid weight gain, which, in turn, has been associated with an increased risk of overweight and obesity later in life [4, 5].

Growth and body composition development contribute to the programming process and are affected by early feeding choices [6]. Compared to breastfed infants, who achieve a rapid increase in fat mass content within the first 4–6 months of age, formula-fed infants show a higher fat-free mass content in the first months of life, with a tendency towards greater adiposity at the 12th month of life. These findings suggest that formula feeding is associated with an alteration in body composition development, which could partially be due to the high protein intake consumed by formula-fed infants and could negatively affect the development of intermediary metabolism and/or long-term appetite regulation [7].

Currently, formulas must meet the permitted protein amounts, which allow a protein range between 1.8 and 3.0 g/100 kcal [8–10]. However, there is no agreement on the appropriate amount of protein in infant formulas [11]. The United States Food and Drug Administration recommends a protein content ranging between 1.8 and 4.5 g/100 kcal, according to the biological quality of protein [12]. Recently, the European Food Safety Authority proposed to lower the maximum protein content of infant formula to 2.5 g/100 kcal. In addition, it has been recommended that formulas with a protein content between 1.80 and 2 g/100 kcal should undergo clinical assessments of their safety and suitability [7].

In this study, we aimed to evaluate the tolerability and safety of a formula with a protein content similar to that of the mature human milk in healthy full-term infants by investigating effects on growth and gastrointestinal tolerability and by identifying adverse effects.

## Methods

### Subjects

All consecutive newborns admitted to the authors’ institution between June 2014 and January 2016 were screened for eligibility. Inclusion criteria for all enrolled infants were as follows: healthy, singleton, full-term infants (gestational age 37/0 to 41/6 weeks), with a birth weight adequate for gestational age (>10th percentile and <90th percentile for gestational age) according to the World Health Organization growth charts (available at http://www.who.int/childgrowth/standards/en/), and aged up to 3 weeks when entering the study. Exclusion criteria were the presence of congenital diseases, chromosomal abnormalities and/or conditions that could interfere with growth, such as brain, metabolic, cardiac and gastrointestinal diseases, perinatal infections, being born to a mother affected by endocrine and/or metabolic diseases, or having a family history of allergic disease.

### Design

We conducted a prospective, controlled, single-blinded randomized trial. All mothers of infants enrolled in the study were encouraged to breastfeed their infants for at least 4 months. Enrolment and randomization occurred concurrently and were performed within 3 weeks after birth. If mothers could not or intended not to breastfeed their infants, the study investigators asked the mothers for their consent to participate in the study. Infants were randomized to receive either formula A or formula B. The composition differences of formula A vs. formula B were in energy (65 vs. 68 kcal/100 mL), protein (1.2 vs. 1.7 g/100 mL), protein-to-energy ratio (1.9 vs. 2.5 g/100 kcal), carbohydrates (8 vs. 7.1 g/100 mL), fat (3.1 vs. 3.5 g/100 mL). The randomization was performed by an independent investigator using a computer-generated randomization list with a random permuted block size of 4. Infants were fed on demand. At enrolment, parents were instructed to record the daily quantities of milk consumed by the infants. Energy and protein daily intakes were then calculated.

To investigate the safety of formula A without any bias, a 4-month intervention period was selected during which the infants were fed only formula.

Anthropometry (weight, length, and head circumference) and body composition were assessed at enrolment, 2 and 4 months of age. Parents were asked to keep a diary on the occurrence of gastrointestinal symptoms or any other symptoms and were contacted every 2 weeks by either clinic visits or phone calls.

The reference group consisted of a cohort of healthy, exclusively breastfed, full-term infants.

### Procedures

#### Growth parameters

Anthropometric and body composition measurements were performed by three medical investigators who were blinded to the allocated treatment. Body weight, length and head circumference were measured according to standard procedures [13]. Weight was measured on an electronic scale accurate to 0.1 g (PEA POD Infant Body Composition System; Cosmed, Concord, CA, USA). Body length was measured to the nearest 1 mm on a Harpenden neonatometer (Holtain, Crymych, UK). Head circumference was measured to the nearest 1 mm using non-stretch measuring tape. Z-score values for age were calculated using the z-score calculator provided by the World Health Organization [WHO Anthro (version 3.2.2, January 2011)].

Weight velocity was calculated as the change in body weight from the weight at study enrolment divided by the time interval from enrolment to the assessment at 4 months [14]. Fat mass and fat-free mass increases (g/day) were also calculated, respectively, as the change in fat mass and fat-free mass content from fat mass and fat-free mass content at study enrolment divided by the time interval from enrolment to the assessments at 2 and 4 months.

Body composition was assessed using an air-displacement plethysmography (PEA POD Infant Body Composition System; COSMED, Italy). A detailed description of the PEA POD’s physical design, operating principles, validation, and measurement procedures is provided elsewhere [15, 16].

#### Gastrointestinal tolerance parameters

The occurrence of spitting up, vomiting and colic, defined as intermittent attacks of abdominal pain when the baby screamed and drew up his/her legs but that abated between episodes, was recorded. Colic was further classified as severe if the episodes occurred more than twice per day. Daily frequency of stool passage was also recorded.

#### Adverse events

Adverse events were assessed based on inquiries to the parents and on daily records. All adverse events were evaluated by the investigator for causal relationship to the study feeding and for severity. An adverse event was defined as any event that was not consistent with the information provided in the consent form or that could not reasonably be expected to accompany the natural history and progression of the subject’s condition throughout the study. Adverse events were considered serious if they were fatal or life-threatening, required hospitalization or surgical intervention, resulted in persistent or significant disability/incapacity or were considered to be medically relevant by the investigator. All other adverse events were categorized as non-serious.

#### Statistical analysis

We hypothesized that the weight gain of the infants fed the formula A would be similar to that of infants fed the formula B. Studying 50 infants per group would permit detection of a difference of 4 g/day ±5.7 standard deviation (95% Confidence Interval: − 1.8; − 6.8), in weight gain through the study, so that the cumulative growth difference would not be lower than the standard deviation for weight (610 g) [17, 18]. Thus, assuming a mean growth velocity of 25 g/day in the infants fed the formula B, the growth of the infants fed the formula A would not be lower than 18.8 g/day or higher than 23.2 g/day.

Breastfeeding group infants were not randomized and constituted the reference group.

Continuous variables were reported as the mean and standard deviation (SD). Categorical variables were reported as numbers or percentages. To test the hypothesis that the weight gain of the infants fed the 1.2 g of protein content formula (formula A) would be similar to that of infants fed the 1.7 g of protein content formula (formula B), differences between groups in measurements of growth and body composition parameters were assessed by analysis of variance. The χ^2^ test was used for comparisons between discrete variables. Bonferroni corrections for multiple comparisons were conducted between the two formula groups and the reference group.

Statistical significance was set at alpha = 0.05. Statistical analyses were conducted using SPSS (Statistical Package for the Social Sciences) version 12 software (SPSS Inc., Chicago, IL, USA).

## Results

### Study population

**Fig. 1:**
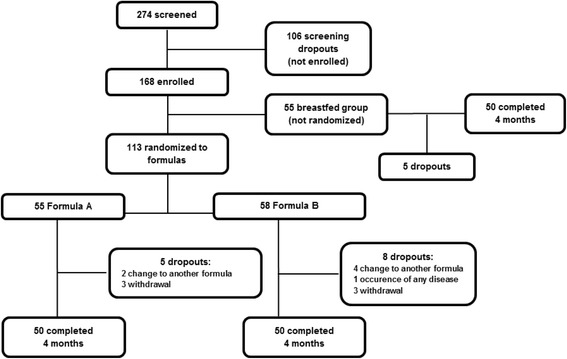
The trial profile

A total of 274 infants were screened between June 2014 and January 2016; 168 infants were enrolled and randomized into one of the two study groups or, if fully breastfed, were included in the breastfeeding group. The mean age at enrolment was 5.3 ± 3.5 days. During the intervention period, 18 infants (11%) dropped out. The trial profile is shown in Fig. 1. No differences in the characteristics at birth or in the growth measurements, when last assessed, were observed between infants who were lost at follow-up and the infants who were evaluated.

The baseline characteristics of the enrolled infants are summarized in Table 1. No differences between the two formula groups and the reference group were found.

**Table 1:** Basic characteristics of the enrolled infants

	Formula A group (*n* = 50)	Formula B group (n = 50)	Breastfeeding group (n = 50)
Gestational age (weeks)	38.6 ± 1.1	38.3 ± 1.0	39.2 ± 1.3
Birth weight (g)	3273.6 ± 381	3137.8 ± 433	3180.6 ± 400
Birth length (cm)	49.6 ± 1.2	49.0 ± 2.1	49.1 ± 2.3
Birth head circumference (cm)	34.2 ± 1.1	33.9 ± 1.3	34.1 ± 1.1
Days at enrolment (n)	5.14 ± 2.2	5.1 ± 3.6	5.3 ± 2.3
Males (n, %)	22 (44)	29 (58)	21 (42)
Caesarean section (n, %)	35 (70)	28 (56)	18 (36)

### Growth and body composition

In Table 2, the anthropometric measurements throughout the study of enrolled infants are shown. Growth parameters were similar between the two formula study groups at each study point. The infants fed formula A and the formula B showed a slightly different growth pattern throughout the study compared to the breastfed infants. At 2 months of life, the infants fed formula A were heavier than the control group, whereas no differences were found at 4 months between the formula fed infants and the breastfed infants. Similar results were found taking into account the z-score values. The length values were similar among the groups at each study point, whereas the head circumference was greater in infants fed formula A and formula B compared to breastfed infants at 2 months. Regarding z-score parameters, infants fed formula A showed a higher value at enrolment compared to the formula B fed infants. Similar length z-scores were found at two and 4 months for all infants enrolled.

**Table 2:** Anthropometric measurements at each study point in enrolled infants

		Formula A group	Formula B group	Breastfeeding group	*P*
Weight (g)	Enrolment	3095.3 ± 333	2999.9 ± 428	2971.8 ± 444	ns
	2 months	5341.5 ± 623	5174.1 ± 621	4999.2 ± 651	^c^: 0.033
	4 months	6856.5 ± 832	6685.9 ± 800	6480.9 ± 823	ns
Weight z-score	Enrolment	−0.32 ± 0.8	− 0.52 ± 1.1	− 0.69 ± 1.0	ns
	2 months	−0.08 ± 0.7	− 0.35 ± 0.9	− 0.55 ± 0.9	^c^: 0.033
	4 months	0.12 ± 0.9	−0.18 ± 0.9	−0.35 ± 0.9	^c^: 0.048
Length (cm)	Enrolment	49.7 ± 1.9	49.1 ± 2.0	49.1 ± 2.3	ns
	2 months	57.1 ± 2.5	56.8 ± 2.8	57.1 ± 2.4	ns
	4 months	63.1 ± 2.2	62.5 ± 2.6	62.8 ± 2.4	ns
Length z-score	Enrolment	−0.38 ± 0.91	−0.51 ± 1.2	−0.55 ± 1.1	^a^: 0.018
	2 months	−0.26 ± 1.1	−0.61 ± 1.2	− 0.32 ± 1.1	ns
	4 months	0.08 ± 0.9	−0.31 ± 1.1	−0.14 ± 0.9	ns
Head circumference (cm)	Enrolment	34.2 ± 1.1	33.9 ± 1.3	34.1 ± 1.1	ns
	2 months	39.1 ± 1.4	39.0 ± 1.6	38.2 ± 1.3	^b^: 0.016 ^c^: 0.009
	4 months	41.5 ± 1.5	41.7 ± 1.6	40.8 ± 1.4	^b^: 0.01
Head circumference z-score	Enrolment	−0.35 ± 0.8	−0.65 ± 1.0	−0.35 ± 0.9	ns
	2 months	0.24 ± 1.0	0.20 ± 1.2	−0.38 ± 0.9	^b^: 0.034 ^c^: 0.017
	4 months	0.50 ± 1.1	0.39 ± 1.0	−0.24 ± 1.0	^b^: 0.020 ^c^: 0.020

^a^: formula B group vs. formula A group

^b^: formula B group vs. breastfeeding group

^c^: formula A group vs. breastfeeding group

At fourth months only infants fed formula A showed a greater head circumference compared to breastfed infants.

The mean weight gain (g/day) throughout the study was not different between the formula A and formula B groups [32.8 ± 6.8 (95% CI: 30.9–34.9) vs. 32.5 ± 6.1 (95% CI: 30.6–34.4), respectively] and from the breastfed infants [30.4 ± 5.4 (95% CI: 28.7–32.0)].

In Table 3, the body composition parameters are reported. No difference was found between the formula groups at any study point. Infants of the formula A and formula B groups showed similar fat mass deposition at each study points compared to the breastfed infants, whereas a different fat-free mass content in comparison to breastfed infants at two and 4 months was detected.

**Table 3:** Body composition parameters of the enrolled infants

		Formula A group	Formula B group	Breastfeeding group	*P*
Fat mass (g)	Enrolment	251.0 ± 132	258.1 ± 127	202.1 ± 119	ns
	2 months	1185.0 ± 274	1122.8 ± 273	1137.1 ± 320	ns
	4 months	1778.4 ± 454	1674.3 ± 357	1772.7 ± 413	ns
Fat mass (%)	Enrolment	7.7 ± 3.6	8.2 ± 3.5	6.5 ± 3.2	ns
	2 months	21.2 ± 3.8	21.3 ± 3.8	22.6 ± 4.1	ns
	4 months	25.9 ± 4.0	25.2 ± 4.3	27.5 ± 4.3	ns
Fat-free mass (g)	Enrolment	2844.3 ± 284	2798.4 ± 357	2765.9 ± 375	ns
	2 months	4177.2 ± 422	4105.6 ± 423	3825.0 ± 414	^b^: 0.01 ^c^: < 0.001
	4 months	5014.8 ± 514	4952.0 ± 493	4565.4 ± 428	^b^: 0.002 ^c^: < 0.001
Fat-free mass (%)	Enrolment	92.0 ± 3.8	91.7 ± 3.7	93.4 ± 3.2	ns
	2 months	78.1 ± 3.2	78.7 ± 3.8	77.4 ± 4.1	ns
	4 months	74.1 ± 4.0	74.8 ± 4.3	72.2 ± 4.6	^b^: 0.033

^a^: formula B group vs. formula A group

^b^: formula B group vs. breastfeeding group

^c^: formula A group vs. breastfeeding group

In Table 4, the fat mass and fat-free mass increase (g/day) during the study period are shown. No difference in fat mass or fat-free mass increase was found between the formula A and formula B groups throughout the study. However, unlike what was observed for the formula B group, the formula A group showed a mean fat-free mass increase value similar to that of the breastfed infants, particularly from enrolment to 2 months.

**Table 4:** Mean fat mass and fat-free mass increase (g/day) during the study period

		Formula A group	Formula B group	Breastfeeding group	*P*
Fat mass increase (g/day)	Enrolment-2 months	17.2 ± 5.6	16.5 ± 5.5	17.1 ± 5.0	ns
	2–4 months	11.5 ± 6.5	10.7 ± 5.5	12.4 ± 8.2	ns
	Enrolment-4 months	13.3 ± 4.1	12.7 ± 3.1	13.7 ± 3.4	ns
Fat-free mass increase (g/day)	Enrolment-2 months	24.6 ± 5.5	29.3 ± 15.3	21.1 ± 7.2	^b^: 0.001
	2–4 months	14.0 ± 6.5	14.3 ± 5.6	13.9 ± 9.3	ns
	Enrolment- 4 months	19.0 ± 3.6	21.5 ± 8.0	16.5 ± 4.5	^b^: 0.001

^a^: formula B group vs. formula A group

^b^: formula B group vs. breastfeeding group

^c^: formula A group vs. breastfeeding group

### Nutritional intakes

In Table 5, the nutritional intakes during the intervention period (2 months and 4 months) for the formula study groups are shown. Infants fed formula B consumed a higher protein intake throughout the study than the infants fed formula A. The energy intake of infants fed formula A was lower at 4 months compare to infants fed formula B.

**Table 5:** Nutritional intakes during the intervention period (2 months and 4 months of life) in the two formula study groups

		Formula A group	Formula B group	*p*
2 months	Numbers of feedings/day	5.3 ± 0.7	5.2 ± 0.8	0.408
	Volume of milk (ml/kg/day)	156.5 ± 25.1	153.0 ± 21.6	0.498
	Energy (kcal/kg/day)	101.7 ± 16.3	104.0 ± 14.7	0.507
	Proteins (g/kg/day)	1.88 ± 0.3	2.60 ± 0.36	< 0.0001
4 months	Numbers of feedings/day	4.7 ± 0.7	4.6 ± 0.6	0.664
	Volume of milk (ml/kg/day)	130.3 ± 16.5	135.6 ± 20.5	0.188
	Energy (kcal/kg/day)	84.7 ± 10.7	92.25 ± 13.9	0.006
	Proteins (g/kg/day)	1.56 ± 1.9	2.31 ± 0.35	< 0.0001

### Gastrointestinal tolerance

In Table 6, data related to gastrointestinal tolerance during the treatment period are shown. No differences in the occurrence of any gastrointestinal symptoms were detected between the formula study groups. Both groups showed a similar daily stool frequency at each study point.

**Table 6:** Gastrointestinal tolerance in the enrolled infants in the two study groups during the study

		Spitting n (%)	Vomiting n (%)	Colic n (%)	Daily stool frequency mean (SD)
2 months	Formula A group	30 (60)	1 (2)	27 (54)	1.6 ± 1.0
	Formula B group	25 (50)	4 (8)	25 (50)	1.5 ± 0.7
4 months	Formula A group	22 (44)	0 (0)	8 (16)	1.4 ± 0.7
	Formula B group	30 (60)	0 (0)	10 (20)	1.6 ± 0.7

### Adverse events

Overall, 12 adverse events occurred in 12 infants. Of these, 3 were categorized as serious. Documented reasons for all adverse events were mostly illnesses that are common during the first year of life (for example, bronchiolitis, gastroenteritis, urinary tract infection, pharyngitis). There were no differences in the occurrence of adverse events during the study between the two study groups.

## Discussion

The results of the current study indicate that feeding formula A (1.9 g/100 kcal) is well tolerated and safe, allowing adequate growth, as demonstrated by the achievement of z-score values of anthropometric parameters close to the median of the reference values [19]. However, it must be noted that although it is adequate, the growth supported by the consumption of formula A appears to be associated with a preferential deposition of fat-free mass rather than fat mass through the study period. Therefore, considering the different pattern of growth that formula fed infants showed compared to that observed in breastfed infants, being formula fed, also with a lower protein content, does not apparently prevent the potential alteration in body composition development described in formula-fed infants [7]. It could be assumed that the major amount of fat-free mass of formula fed infants could be explained by formula’s macronutrients content. Kashyap et al. [20], in a study conduct on premature infants, demonstrated that there was a significant positive correlation between the protein storage and carbohydrate intake that was not related to fat intake. In the present study, we did not analyse the macronutrient content of human milk of the reference group. It has been demonstrated that the human milk mean carbohydrate and fat content in the first 8 weeks postpartum is 6.2 ± 0.9 g/dL and 4.1 ± 0.7 g/dL respectively [21]. Therefore, the major amount of fat-free mass observed in formula fed infants compared to reference group could be explained by the higher carbohydrate intake, which could facilitate the protein storage. Conversely, it could also be speculated that the lack of an effect on modulating the development of body composition in the first months of life between the two formula groups could be partially explained by the relatively limited duration of the intervention. When considering the fat-free mass increase through the study, the infants in the formula A group showed a mean fat-free mass increase similar to that of breastfed infants, particularly between enrolment and 2 months. Furthermore, the lack of difference in absolute fat-free mass content between the two study formula groups at the end of the study could be because the study was actually powered on weight gain rather than fat-free mass increase.

The results of the present study concerning the safety of the consumption of a low-protein formula in healthy term infants are consistent with previously published data. Abrams et al. [22] conducted a systematic review, including 6 studies, and concluded that feeding a low-protein formula leads to adequate growth during infancy and early childhood. Patro-Gołąb et al. [11] investigated the evidence available in the literature related to the effects of different protein formula contents (range 1.1–3.2 g/100 mL) on infants’ growth. The authors reported that the studies included in their systematic review evaluated only the short-term effects on growth, from three to 5 months of age, without finding any modification of infants’ growth with regard to length and weight gains and BMI, regardless of protein formula concentration.

To our knowledge, there is a paucity of studies investigating the safety of feeding a low-protein formula, including body composition assessment. The CHOP study demonstrated that the consumption of a formula with a protein ratio of 1.77 g/100 kcal during the first year of life promotes ponderal growth similar to that of breastfed infants and lower than that of infants fed a formula with a high protein-energy intake (2.9 g/100 kcal) and is associated with a lower risk for being obese at school age [4, 5]. In agreement with our results, the authors did not find any significant difference in the fat mass or fat-free mass content at 6 months of age [23]. However, the authors reported that a higher protein intake in formula-fed infants appears to promote visceral fat mass accumulation at prepubertal age, which is a known risk factor for adverse metabolic and health consequences, whereas the deposition of subcutaneous fat appears not to be affected [24]. It could be then hypothesized that although no significant difference was detected in the total fat-free mass content between the formula study groups in the present study, the consumption of a low protein intake in this critical time window could contribute to the developmental programming of fat mass distribution in later life.

The main strength of this work is represented by the fact that this study is a prospective, longitudinal study, conducted on a relatively large number of infants. However, it must be taken into consideration that both the intervention and the follow-up period were relatively short; as a result, the potential cumulative effect of consuming a low-protein formula in early infancy on growth and body composition in the medium to long term has not been assessed.

## Conclusion

On the basis of the present findings, the consumption of a low-protein formula during the first months of life appears to be safe and to allow adequate growth. Additional studies and a longer follow-up are needed to gain further insight into the effect that different protein formula contents have on body composition development.

## Abbreviations

CHOP: European Childhood Obesity Trial Study Group
SD: Standard deviation

## References

[1] Godfrey KM, Costello PM, Lillycrop KA (2016). Development, epigenetics and metabolic programming. Nestle Nutr Inst Workshop.

[2] Mameli C, Mazzantini S, Zuccotti GV (2016). Nutrition in the first 1000 days: the origin of childhood obesity. Int J Environ Res Public Health.

[3] American Academy of Pediatrics (2012). Section on breastfeeding: breastfeeding and the use of human milk. Pediatrics.

[4] Koletzko B, von Kries R, Closa R, Escribano J, Scaglioni S, Giovannini M, Beyer J, Demmelmair H, Gruszfeld D, Dobrzanska A, Sengier A, Langhendries JP, Rolland Cachera MF, Grote V (2009). European childhood obesity trial study group: lower protein in infant formula is associated with lower weight up to age 2 y: a randomized clinical trial. Am J Clin Nutr.

[5] Weber M, Grote V, Closa-Monasterolo R, Escribano J, Langhendries JP, Dain E, Giovannini M, Verduci E, Gruszfeld D, Socha P, Koletzko B (2014). European childhood obesity trial study group: lower protein content in infant formula reduces BMI and obesity risk at school age: follow-up of a randomized trial. Am J Clin Nutr.

[6] Wells JC (2012). Body composition in infants: evidence for developmental programming and techniques for measurement. Rev Endocr Metab Disord.

[7] Gale C, Logan KM, Santhakumaran S, Parkinson JR, Hyde MJ, Modi N (2012). Effect of breastfeeding compared with formula feeding on infant body composition: a systematic review and meta-analysis. Am J Clin Nutr.

[8] European Union Commission Directive 2006/141/EC. Infant formulae and follow-on formulae. Official Journal of the European Communities 2015. Available from: http://eur-lex.europa.eu/legal-content/EN/ALL/?uri=CELEX%3A32006L0141.

[9] EFSA Panel on Dietetic Products, Nutrition and Allergies (NDA). Scientific Opinion on the essential composition of infant and follow-on formulae. EFSA Journal. 2014;12:3760.

[10] Codex Alimentarius International Food Standards. Standard for infant formula and formulas for special medical purposes intended for infants. 2014. Available from: www.codexalimentarius.org.

[11] Patro-Gołąb B, Zalewski BM, Kouwenhoven SM, Karaś J, Koletzko B, Bernard van Goudoever J, Szajewska H (2016). Protein concentration in milk formula, growth, and later risk of obesity: a systematic review. J Nutr.

[12] Food and Drug Administration Department of Health and Human Services. Code of Federal Regulations. Subchapter B-Food for human consumption, pat 107: Infant formula-Subpart D: Nutrient Requirements.2017. Available from www.fda.gov.

[13] Agostoni C, Grandi F, Giannì ML, Silano M, Torcoletti M, Giovannini M, Riva E (1999). Growth patterns of breast fed and formula fed infants in the first 12 months of life: an Italian study. Arch Dis Child.

[14] Piemontese P, Giannì ML, Braegger CP, Chirico G, Grüber C, Riedler J, Arslanoglu S, van Stuijvenberg M, Boehm G, Jelinek J, Roggero P (2011). MIPS 1 working group: tolerance and safety evaluation in a large cohort of healthy infants fed an innovative prebiotic formula: a randomized controlled trial. PLoS One.

[15] Roggero P, Giannì ML, Amato O, Piemontese P, Morniroli D, Wong WW, Mosca F (2012). Evaluation of air-displacement plethysmography for body composition assessment in preterm infants. Pediatr Res.

[16] Urlando A, Dempster P, Aitkens S (2003). A new air displacement plethysmograph for the measurement of body composition in infants. Pediatr Res.

[17] Roggero P, Giannì ML, Orsi A, Piemontese P, Amato O, Liotto N, Morlacchi L, Taroni F, Fields DA, Catalano PM, Mosca F (2010). Quality of growth in exclusively breast-fed infants in the first six months of life: an Italian study. Pediatr Res.

[18] Fomon SJ, Ziegler EE, Nelson SE, Rogers RR, Frantz JA (1999). Infant formula with protein-energy ratio of 1.7 g/100 kcal is adequate but may not be safe. J Pediatr Gastroenterol Nutr.

[19] WHO Multicentre Growth Reference Study Group (2006). WHO child growth standards: length/height-for-age, weight-for-age, weight-for-length, weight-for-height and body mass index-for-age: methods and development.

[20] Kashyap S, Towers HM, Sahni R, Ohira-Kist K, Abildskov K, Schulze KF (2001). Effects of quality of energy on substrate oxidation in enterally fed, low-birth-weight infants. Am J Clin Nutr.

[21] Bauer J, Gerss J (2011). Longitudinal analysis of macronutrients and minerals in human milk produced by mothers of preterm infants. Clin Nutr.

[22] Abrams SA, Hawthorne KM, Pammi M (2015). A systematic review of controlled trials of lower-protein or energy-containing infant formulas for use by healthy full-term infants. Adv Nutr.

[23] Escribano J, Luque V, Ferre N, Mendez-Riera G, Koletzko B, Grote V, Demmelmair H, Bluck L, Wright A, Closa-Monasterolo R (2012). European childhood obesity trial study group: effect of protein intake and weight gain velocity on body fat mass at 6 months of age: the EU childhood obesity Programme. Int J Obes.

[24] Gruszfeld D, Weber M, Gradowska K, Socha P, Grote V, Xhonneux A, Dain E, Verduci E, Riva E, Closa-Monasterolo R, Escribano J, Koletzko B (2016). European childhood obesity study group: association of early protein intake and pre-peritoneal fat at five years of age: follow-up of a randomized clinical trial. Nutr Metab Cardiovasc Dis.

