# Correction to: Clinical evaluation of two different protein content formulas fed to full-term healthy infants: a randomized controlled trial

**DOI:** 10.1186/s12887-018-1109-8

**Published:** 2018-04-12

**Authors:** Nadia Liotto, Anna Orsi, Camilla Menis, Pasqua Piemontese, Laura Morlacchi, Chiara Cristiana Condello, Maria Lorella Giannì, Paola Roggero, Fabio Mosca

**Affiliations:** 1 Fondazione I.R.C.C.S. Ca Granda Ospedale Maggiore Policlinico, Neonatal Intensive Care Unit Department of Clinical Science and Community Health, University of Milan, Milan, Italy; 2 Centro di Nutrizione a Partenza neonatale, Clinica Mangiagalli, Via Della Commenda, 12, 20122 Milan, Italy

## Correction

Following the publication of the original article [1], it was brought to our attention that the authors’ names and surnames were erroneously interchanged.

The corrected authors’ names and surnames are shown below:

Nadia Liotto

Anna Orsi

Camilla Menis

Pasqua Piemontese

Laura Morlacchi

Chiara Cristiana Condello

Maria Lorella Giannì

Paola Roggero

Fabio Mosca

These have also been included in the author list of this ‘Correction’ and updated in the original article [1].
